# Common clinical pattern of antineutrophil cytoplasmic antibody -associated vasculitis: An experience from a multicenter study in Saudi Arabia

**DOI:** 10.15537/smj.2023.44.5.20220818

**Published:** 2023-05

**Authors:** Jumanah J. Alfuwayris, Amal M. Almulhim, Mohammed A. Almansour, Marzouq A. Albadi, Ibrahim A. Alhomood, Faisal Alblewi, Abdullah U. Althemery

**Affiliations:** *From the Department of Rheumatology (Alfuwayris), Ministry of the National Guard – Health Affairs, King Abdullah International Medical Research Center, from the Department of Rheumatology (Alfuwayris), King Saud bin Abdulaziz University for Health Sciences; from Department of Rheumatology, King Fahad Medical City (Almulhim, Almansour, Alhomood, Alblewi); from Rheumatology Division (Albadi), Security Forces Hospital, Riyadh; and from Department of Clinical Pharmacy (Althemery), College of Pharmacy, Prince Sattam bin Abdulaziz University, Al-Kharj, Kingdom of Saudi Arabia.*

**Keywords:** antineutrophil cytoplasmic antibody, AN CA-associated vasculitis, autoimmune disorder, Saudi Arabia, clinical manifestations

## Abstract

**Objectives:**

To understand the most common type and clinical manifestations of associated vasculitis (AAV) in the Saudi Arabia.

**Methods:**

This retrospective study was conducted at King Fahad Medical City and the Security Forces Hospital Program, Riyadh, Saudi Arabia, between January 2014 and May 2022. Patients aged ≥18 years were included in the study and diagnosed based on clinical manifestations, serology, or histopathology according to the EMA algorithm. Univariate analysis was carried out to compare different groups; a series of independent samples t-tests was applied for continuous data.

**Results:**

A total of 53 patients were enrolled: eosinophilic granulomatosis with polyangiitis (EGPA), granulomatosis with polyangiitis (GPA), and microscopic polyangiitis (MPA). Overall, proteinase-3 was the most prevalent (52.8%), and myeloperoxidase, myeloperoxidase MPO was the least prevalent antineutrophil cytoplasmic antibody (ANCA)-type (18.9%) among patients; other patients showed negative ANCA test results. The clinical manifestations differed significantly between EGPA and GPA groups in pulmonary, neurological, cardiological, and renal signs and symptoms (*p*<0.05); there was a higher incidence of the former 3 in the EGPA group. Although upper airway was predominant in all groups, there was no statistical difference between both groups.

**Conclusion:**

This study validated international reports on AAV clinical manifestations in the Saudi population. The GPA was associated with more upper airway and pulmonary signs and symptoms. Further investigation is needed to understand the treatments and quality of life of patients with AAV.

**A**ntineutrophil cytoplasmic antibody (ANCA)-associated vasculitis (AAV) is an autoimmune disorder that affects small- and, to a lesser degree, medium-sized vessels. The AAV encompasses 3 disease phenotypes: granulomatosis with polyangiitis (GPA), microscopic polyangiitis (MPA), and eosinophilic granulomatosis with polyangiitis (EGPA).^1^ These diseases predominantly affect the small blood vessels, causing necrotizing vascular inflammation in different organs with a wide spectrum of manifestations.^2^

The annual incidence of AAV has been increasing over the last years, with an overall rate ranging from 13.1–20.9% per million in different countries.^3^ These disorders tend to occur equally in both genders, with a peak age between 65 and 74 years at diagnosis.^4^ Individuals diagnosed with AAV can present with severe clinical symptoms that may threaten their lives, for example, alveolar hemorrhage and rapidly progressive glomerulonephritis, while other patients may complain of a less severe clinical picture involving any part of the upper respiratory tract.^5^ Two fluorescence patterns of ANCAs distinguished by indirect immunofluorescence (IIF) have been associated with AAV: the cytoplasmic staining pattern (C-ANCA) and perinuclear staining pattern (P-ANCA). Most patients with C-ANCA have ANCAs directed against proteinase-3 (PR3), whereas most patients with P-ANCA have ANCAs directed against myeloperoxidase (MPO), identified using antigen-specific enzyme-linked immunosorbent assay (ELISA).^6^ More recently, several studies have suggested that predicting clinical outcomes is more accurate when AAV subsets are classified into PR3-AAV and MPO-AAV by the specific ANCA profile.^7^ The MPO-ANCA recurrence/persistence identifies patients with a lower potential for renal recovery and a higher risk of kidney failure, whereas PR3-ANCA recurrence/persistence identifies patients with a greater relapse risk.^8^ Although any tissue can be involved in AAV, the upper and lower respiratory tract and kidneys are most commonly and severely affected, ear-nose-throat (ENT) and ocular involvement are more frequent in GPA, and neurological and cardiovascular involvement are more frequent in EGPA.^9-11^ Patients with AAV-related renal injury have a higher rate of early mortality.^12^

End-stage kidney disease (ESKD) and premature death remain common among patients with ANCA–associated vasculitis who present with reduced kidney function or pulmonary hemorrhage.^1^ Poor outcomes are attributed to a delay in diagnosis and use of treatments that have a slow onset of action, incomplete efficacy, and toxic effects.^13^

The prognosis of patients with AAV is a crucial issue due to the accumulating damage caused by both the disease activity and treatment toxicity.^14^

The characteristics of patients with AAV in Saudi Arabia require further exploration.^4^ Numerous studies have been conducted on AAV in Saudi Arabia, many of which have explored the manifestations of these disorders.^15^ Research into these disorders is not common; as such, we sought to understand the most common type and clinical manifestations of AAV and to gain additional background knowledge to publish more studies in this field.

## Methods

This retrospective study was carried out at King Fahad Medical City (KFMC) and the Security Forces Hospital Program (SFH), Riyadh, Saudi Arabia, between January 2014 and May 2022. The KFMC and SFH are referral hospitals with advanced health care facilities with a bed capacity of 1200 and 1068 beds, respectively.

The rheumatology clinic was the major clinic investigated. However, other facilities and subspecialty units, including rheumatology, nephrology, pulmonology, ENT, ophthalmology, neurology, intensive care unit, cardiology, and dermatology units, needed for the diagnosis and treatment of patients with AAV were available.

The medical records of patients with AAV and study data were electronically extracted from a hospital database dedicated to patients who used the rheumatology clinic.

The participants’ demographic data at the time of presentation included patient age, gender, presenting clinical manifestations, and laboratory variables including complete blood count, absolute eosinophil count, inflammatory markers (erythrocyte sedimentation rate [ESR], C-reactive protein [CRP]), creatinine, albumin, urine red blood cells (RBCs), 24 hours urine protein, and anti-PR3/anti-MPO antibodies (analyzed using ELIZA).

Patients aged ≥18 years were included in the study and diagnosed based on clinical manifestations, serology, and/or histopathology according to the European Medicine Agency algorithm by Watts et al.^16^ Moreover, patients with positive ANCA results secondary to causes other than vasculitis were excluded from the study.

The study was approved by King Fahad Medical City and the Security Forces Hospital Program (IRB registration numbers KACST, KSA: H-01-R-012, H-01-R-069). Informed or written consent was not required for this retrospective study. All patient data were secured, and only the principal investigator had access to them.

### Statistical analysis

Univariate analysis was conducted to compare different groups; a series of independent samples t-tests was applied for continuous data, and Fisher’s exact test was used for categorical data. *P*-value<0.05 was considered statistically significant. All analyses were conducted using R (R Foundation for Statistical Computing, Vienna, Austria), a language and an environment for statistical computing.

## Results

**Figure 1:**
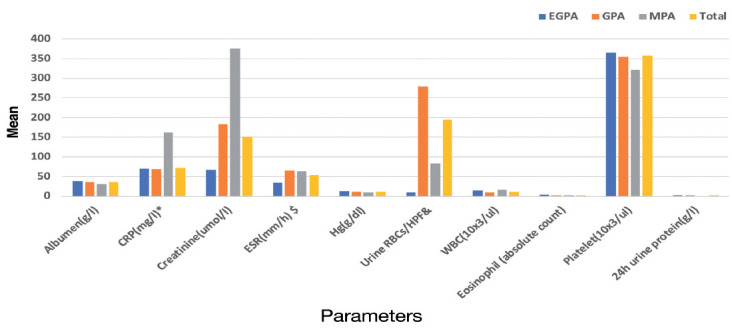
- Means for laboratory parameters. CRP: C-reactive protein, ESR: erythrocyte sedimentation rate, Hg: hemoglobin, RBCs: red blood cells, HPF: high power field, WBC: white blood cell, EGPA: eosinophilic granulomatosis with polyangiitis, GPA: granulomatosis with polyangiitis, MPA: microscopic polyangiitis. *CRP was not needed for 2 patients with GPA. ^$^ESR was not needed for one patients with GPA, ^&^Urine samples were not collected from 4 patients with GPA and 4 patients with EGPA.24h urine protein was collected from only 1 patient: 4.9 g/L.

A total of 53 patients who fulfilled the inclusion criteria were included in the study: 18 with EGPA, 33 with GPA, and 2 with MPA. The average age of patients was 42.81±13.67 years, and there were more females (62.3%) than males (37.8%). Overall, PR3 was the most prevalent ANCA type (52.8%), MPO was less frequent (18.9%), and the remaining patients had negative ANCA tests (28.3%). The most prevalent signs and symptoms were ENT/mucus (86.8%), followed by pulmonary (81.1%), musculoskeletal (MSK) (47.2%), constitutional (43.4%), renal (35.9%), and neurological (28.3%) (Figure 1 & Table 1).

**Table 1:** - Frequencies for categorical paramters (variables).

Variables	EGPA	GPA	*P*-value	Total	MPA	Total
	n (%)			n (%)		
Number of patients	18	33		51	2	53
Average age (years)	40.17 (13.1)	43.61 (14.0)	0.39	42.39 (13.6)	53.50 (13.4)	42.81 (13.7)
Females	11 (61.1)	20 (60.6)	0.24	31 (60.8)	2 (100)	33 (62.3)
***ANCA type***			<0.0001			
MPO	4 (22.2)	4 (12.1)		8 (15.7)	2 (100)	10 (18.9)
Negative	14 (77.8)	1 (3.1)		15 (29.4)	0 (0)	15 (28.3)
PR3	0 (0)	28 (84.9)		28 (54.9)	0 (0)	28 (52.8)
***Systems***						
Upper airway	18 (100)	27 (81.8)	0.08	45 (88.2)	1 (50.0)	46 (86.8)
Pulmonary	18 (100)	24 (72.7)	0.02	42 (82.4)	1 (50.0)	43 (81.1)
Musculoskeletal	8 (44.4)	17 (51.5)	0.77	25 (49.0)	0 (0)	25 (47.2)
Constitutional	5 (27.8)	17 (51.5)	0.14	22 (43.1)	1 (50.0)	23 (43.4)
Renal	1 (5.6)	16 (48.5)	0.002	17 (33.3)	2 (100)	19 (35.9)
Neurology	11 (61.1)	3 (9.1)	0.001	14 (27.4)	1 (50.0)	15 (28.3)
Cutaneous	5 (27.8)	5 (15.2)	0.16	10 (19.6)	0 (0)	10 (18.9)
Cardiology	5 (27.8)	1 (3.1)	0.02	6 (11.8)	0 (0)	6 (11.3)
Gastroenterology	3 (16.7)	3 (9.1)	0.65	6 (11.8)	0 (0)	6 (11.3)
Ocular	1 (5.6)	4 (12.1)	0.64	5 (9.8)	0 (0)	5 (9.4)

EGPA: eosinophilic granulomatosis with polyangiitis, GPA: granulomatosis with polyangiitis, MPA: microscopic polyangiitis, ANCA: antineutrophil cytoplasmic antibody, MPO: myeloperoxidase, PR3: proteinase-3

Laboratory investigations showed that patients with AAV commonly presented with high levels of inflammatory markers, anemia, urine RBCs, and renal affections (Figure 1).

Comparisons of the mean (M) patient characteristics between patients with EGPA and those with GPA with associated standard deviations (SD) are shown in Figure 1. The GPA group had higher creatinine and ESR averages than the EGPA group as follows: (182.61±236.48), (67.17±23.45), *p*=0.01; (63.94±37.09), (33.89±28.33), *p*=0.01, respectively. Patients with GPA showed a significantly lower average hemoglobin level (10.61±2.69) than those with EGPA (12.90±1.92), *p*=0.001. Results of 24 h urine protein were not significantly different between both GPA and EGPA group, (0.57±0.85) and (0.22±0.15), respectively, *p*=0.05, which can be attributed to limited data in both groups.

Table 1 shows the comparison of the frequencies of patient characteristics between patients with EGPA and GPA. Over two-thirds of patients with EPGA showed negative results on the ANCA test, while most patients with GPA were PR3 positive; thus, the relationship between the ANCA test and the subtypes of vasculitis was significant, X^2^=38.15, *p*<0.0001. All patients with EPGA reported the presence of pulmonary-related signs or symptoms, while two-thirds of the patients with GPA reported these symptoms, X^2^=5.96, *p*=0.02. Forty-eight percent of patients with GPA had renal signs and symptoms, while 5.6% with EGPA had renal issues, X^2^=9.66, *p*=0.002. Neurological and cardiac manifestations were significantly more frequent in patients with EGPA than in those with GPA (neurology: X^2^=15.83, *p*=0.001; cardiac: X^2^=6.87, *p*=0.02).

**Figure 2:**
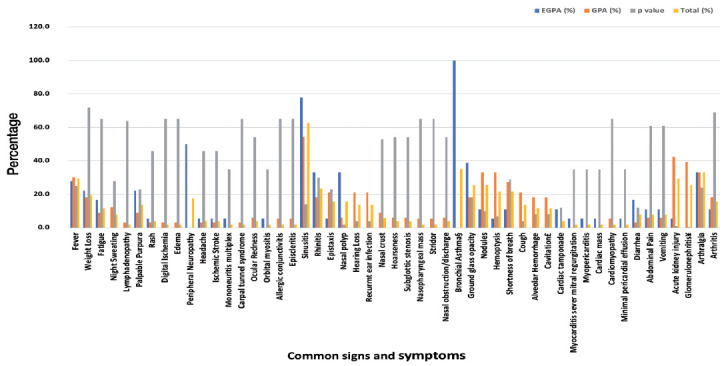
- Common signs and symptoms in patients with eosinophilic granulomatosis with polyangiitis (EGPA) and GPA. EGPA: eosinophilic granulomatosis with polyangiitis, GPA: granulomatosis with polyangiitis. ^§^8 of 18 patients with adult-onset asthma and 4 of 18 with refractory asthma. ^¥^based on renal biopsy: (6) GPA patient with pauci-immune glomerulonephritis [4 of them with crescent], (1) GPA with rapid progressive glomerulonephritis, (1) GPA with pauci-immune and IgA deposit glomerulonephritis, (1) GPA with pauci-immune and focal segmental glomerulonephritis, (1) GPA with focal necrotizing proliferation glomerulonephritis with IgA deposits, (1) GPA with focal necrotizing glomerulonephritis, (2) GPA with focal segmental glomerulonephritis, (1) GPA with fibrous crescent, sclerosed glomerular tuft, and severe vascular changes. Renal biopsy was not performed in 2 GPA and 1 EGPA patients with renal manifestations of acute kidney injury, hematuria, or proteinuria.

Comparison between common signs and symptoms in patients with EGPA and GPA is shown in Figure 2. The most reported sign and symptom was sinusitis, which was observed in 77.8% patients in the EGPA group and 54.5% in the GPA group; however, the relationship between sinusitis and the distribution of vasculitis type was not significant, X^2^=2.69, *p*=0.14. Approximately 33% of patients with EGPA and 6% of patients with GPA developed nasal polyps, showing a significant association between nasal polyps and vasculitis type, X^2^=6.55, *p*=0.02. In terms of renal manifestations, 42.42% of patients with GPA and 5.6% with EGPA had acute kidney injury, X^2^=7.63, *p*=0.01.

Two patients were diagnosed with MPA (Figure 1 & Table 1) and were aged 44 years and 63 years. Both patients were female and had MPO in the ANCA test. Patients with MPA displayed signs and symptoms related to constitutional, neurological, ENT, pulmonary, and renal health. No signs or symptoms related to MSK, cutaneous, cardiology, GI, or ocular health were reported.

## Discussion

The AAV are a collection of relatively rare autoimmune diseases of unknown cause, characterized by inflammatory cell infiltration that causes necrosis of blood vessels. The clinical spectrum of AAV is broad; therefore, its presentation can be quite varied, ranging from a skin rash to fulminant multisystem disease.^15^

While lung and renal involvement are typical manifestations of both GPA and MPA, EGPA usually shows paranasal sinus and lung involvement, as well as a history of bronchial asthma. The EGPA is frequently associated with cardiac disease and peripheral neuropathy.^17^

In this multicenter retrospective study, we described the clinical features and patient characteristics of each ANCA-associated subtype and conducted an analysis to compare the GPA and EGPA groups. The MPA patients were not included in the univariate comparison due to a small sample size.

The study demonstrated that GPA was the most common subtype, with middle aged female predominantly described, which is in accordance with recent data.^4^ Although patients with EGPA and MPA were negative for PR3, our results showed that PR3 was more prevalent than MPO. From a global viewpoint, the findings from Saudi Arabia shared a common trend with reports from North America and Europe, where there was a clear distinction between age and frequency of AVV type and age. In addition, EGPA is more prevalent in younger persons, whereas microscopic polyangiitis and GPA are more prevalent in older patients. Moreover, the prevalence of AAV type affects both sexes equally.^18^

The upper airway is the most commonly affected system in AAV, followed by the pulmonary system. In the subgroup analysis, the frequency of constitutional symptoms (fever) and renal involvement (AKI/glomerulonephritis) were higher in patients with GPA, while cardiac/neurologic (cardiac tamponade and peripheral neuropathy, respectively) symptoms were more common in patients with EGPA. These clinical manifestations are reported locally and internationally. Almaani et al^19^ at Ohio State University summarized international reports of the clinical presentations of ANCA-associated vasculitis, and their final list matches our results.

For local studies there were 2 retrospective reports from Saudi Arabia, Al Arfaj et al.^20^ (examined the clinical manifestations and outcomes of AAV at a tertiary center in Saudi Arabia) and Alahmari et al.^4^ (examined the clinical profile, relapse rate, and disease-related complications among patients with AAV at a tertiary hospital in Saudi Arabia), were published in 2018 and 2021, respectively. These studies and the present study showed that GPA was the most common ANCA subtype, while MPA was the least common. PR3 was more frequently reported than MPO, with the difference in that all MPA patients in Al Arfaj et al. were PR3 positive compared to all MPA patients in this study who were MPO positive. In spite of the results of these studies that showed pulmonary and renal systems as the most commonly affected organs, the present study showed that the upper airway was the most commonly reported systemic involvement, followed by the pulmonary system.

Similar to the study by Al Arfaj et al^20^ and previous reports from China,^21^ fever was significantly more common in patients with GPA than in those with EGPA. Ocular involvement was more frequent in patients with GPA than in those with MPA or EGPA. In line with our results, bronchial asthma was observed in all patients with EGPA in 2 international studies.^21,22^

In the present study, the most commonly manifested rash was palpable purpura, and this is similar to the result of a study by Al Arfaj et al,^20^ although it was more observed in patients with GPA in Al Arfaj et al^20^ compared to patients with EGPA in our study. Neurological involvement was significantly higher in the EGPA group than in the GPA group. In addition, none of the patients with MPA had ocular, cutaneous, cardiac, MSK, or gastrointestinal manifestations, similar to the results of Al Arfaj et al.^20^ However, our study differed from Al Arfaj et al^20^ in that our findings showed the involvement of neurologic manifestation.

### Study limitations

This study had some limitations due to its retrospective nature, including rare disease with a small sample size and insufficient/incomplete data, which may have led to non-significant differences observed in the results, and this may affect the results of this study. Due to the small sample size of patients with MPA, which affects significant statistics, there was limited comparison between MPA and GPA and EGPA patients. In addition, the generalization of the results is not possible.

In conclusion, the GPA and PR3 were the most common ANCA subtypes, and the upper airway and pulmonary involvement were the most predominant clinical manifestations. The ANCA subtype estimated locally was similar to that previously published internationally, with little difference reported in clinical manifestations. More studies with different designs and larger sample sizes are needed to fill the research gaps in ANCA-associated vasculitis in the Kingdom of Saudi Arabia and the Middle Eastern region.
